# Seismic seiche-related oscillations in Lake Biwa, Japan, after the 2011 Tohoku earthquake

**DOI:** 10.1038/s41598-022-23939-7

**Published:** 2022-11-11

**Authors:** Maho Iwaki, Takashi Toda

**Affiliations:** 1 Department of Civil Engineering and Architecture, National Institute of Technology (NIT), Maizuru College, 234 Shiroya, Maizuru City, Kyoto 625-8511 Japan; 2 Lake Biwa Museum, Oroshimo, Kusatsu City, Shiga 525-0001 Japan

**Keywords:** Limnology, Natural hazards, Ocean sciences, Solid Earth sciences

## Abstract

Seismic seiche-related oscillations caused by Rayleigh waves from large earthquakes are yet to be explored and elucidated comprehensively, then need to accumulate continuously. Herein, we investigated water level fluctuations in Lake Biwa of Japan from surface seiches following the 2011 Tohoku earthquake. Lake Biwa is the largest freshwater resource in Japan, and a small change in its water level can affect local ecosystems. Field observations were conducted during 2010–2012 using a water level gauge with a 1 mm resolution and 2 min data sampling interval. Fast Fourier transform and maximum entropy methods were used for data spectral analysis to distinguish the effects of inherent oscillations on water levels generated by the earthquake. We considered that water level changes were influenced by long-period Rayleigh waves. We observed a wave with a 3.08–3.10 h duration, which was close to the duration determined for the Rayleigh waves (3.08 h). The 3.08–3.10 h wave was caused by forced oscillation of Rayleigh waves characterised by a frequency close to the natural frequency and excited by the earthquake. Overall, our findings suggest that water level fluctuations can be good indicators of high-magnitude earthquakes.

## Introduction

Lake Biwa is the largest freshwater resource in Japan, with a surface area of 670.25 km^2^ and a maximum depth of 104.1 m^1^. The northern basin is wide and deep, whereas the southern basin is narrow and shallow. More than 450 rivers and streams flow directly into the lake; however, there is only one natural outlet (Seta River) and two artificial canal outflows that supply water to the city of Kyoto. Moreover, a third outflow is used for hydropower generation. A 1 cm change in the water level of Lake Biwa corresponds to a volume of approximately 6.7 × 10^6^ m^3^. This implies that even a small change in the water level might severely affect local ecosystems, particularly in the shoreline regions^2^. Lake water level fluctuations are essential for the survival of numerous species that have evolved life cycles synchronised with these fluctuations and may be essential for a range of ecosystem services; for example, the changes in the water level of lakes are important for the littoral zone ecosystem and the spawning habitats of endemic fish species^2–5^. In addition, the sensitivity (reaction) of lakes to seismic waves differs depending on the size of the lake. Therefore, it is important to understand the correspondence between seismic waves and the observed seismic waves in lakes of various sizes. Some studies have focused on seismicity–lake-level feedback; Heki^6^ studied the seasonal variation in earthquakes that occurred in Japan, and Ueda and Kato^7^ and Xue et al.^8^ focussed on the seismicity variations in San-in district and Lake Biwa. These papers discuss whether lake-level changes are caused by seismicity or whether they lead to changes in seismicity.

A seiche refers to the periodic oscillation of the surface water of lakes and other closed or half-closed water basins and is typically characterised by standing wave properties. In Japan, the first studies of seiches were conducted at Lake Ashinoko in 1891^9^. Nakamura and Honda^10^ have described seiches in Japanese lakes, including Lake Biwa, and provided detailed technical drawings of the changes in water levels. In response to studies indicating a relationship between the Sanriku tsunami of 1896 and seiches in harbours, several surveys were conducted in major ports and harbours in Japan from 1903 to 1906, which have been compiled into a report by Honda et al.^11^.

Over the past few decades, the surface seiches of Lake Biwa have gained increased research interest. Imasato^12–15^ observed water level fluctuations and developed a numerical simulation model of the seiches from which he identified five dominant seiche modes with periods of 255.5, 79.8, 69.1, 38.7 and 31.9 min. These values were corroborated by field observations that defined the dominant modes as 249.6, 74.1, 65.7, 39.7 and 32.1 min, respectively^16^.

Using the field records from 1730, Forel^17^ showed that seiches could be induced by earthquakes. These earthquake-induced seiches became known as seismic seiches. McGarr and Vorhis^18^ reported seismic seiches generated by the March 1964 Alaskan earthquake. Berninghausen^19–21^ reviewed the relationship between tsunamis and seismic seiches and provided multiple examples, such as the eastern Atlantic south of the Bay of Biscay, Indian Ocean, and Southeast Asia. Using examples from Lake Tahoe, USA, Ichinose et al.^22^ illustrated that local earthquakes beneath a lake have the potential to cause a tsunami, thereby inducing seismic seiches within the lake. Barberopoulou et al.^23^ showed that both seismic waves and seiches occurred in response to the 2002 Alaskan earthquake. Barberopoulou^24^ also investigated large seismic wave motions and various scenarios of seiche generation during strong shaking events in Lake Union, USA. Utsu^25^ reported that under the influence of large earthquakes, an entire body of water within a lake or bay can be agitated by long-period surface waves, thereby leading to free vibrations within the water and resulting in a seiche. Following the 9.0 magnitude Lisbon earthquake of 1755, seismic seiches were observed in the lakes and bays of northern Europe up to 3000 km away from the epicentre^26^. In this case, water level fluctuations of a maximum of 1 m were recorded.

Meanwhile, the undersea volcano Funga Tonga Funga Haapai erupted at around 17:00 (UTC + 13) on 15 January 2022^27^. Tidal levels increased and were observed to be over 80 cm near Nuku’alofa, the capital of Tonga, 70 km south of the eruption point, but they were smaller at tidal observation points along the 8000 km to Japan. However, on the Japanese coast, a maximum tidal level increase of approximately 1 m was observed, causing damage to aquaculture facilities and capsizing approximately 30 ships^28^. The Japan Meteorological Agency (JMA)^28^ suggested that even in the distant coastal areas of Japan, water levels rose, and the Lamb waves damaged public properties before the tsunamis. Under normal circumstances, when the phase velocity of the excited ocean long wave coincides with the movement of the atmospheric disturbance, it resonates and increases in amplitude^28^. However, the report showed that this is not the only case, and that it is important to note that resonance can occur even when the velocities of the ocean gravity and pressure waves do not exactly match, and that if this condition persists for a long time, the tidal level change can also be substantial^28^. Thus, even if the physical phenomenon occurs at a significant distance, large earthquakes or volcanic explosions can cause various wave effects, and continuous recording of such oscillations even in large lakes (large bodies of water on land) would allow a discussion of long-period waves; however, only a few examples of such phenomena have been recorded.

Surface waves propagate around the Earth, with Rayleigh waves exhibiting exceedingly high amplitudes^29,30^. On 26 December 2004, a mega earthquake with a magnitude > 9.0 occurred off the coast of Sumatra. Seismographs revealed that eight revolutions of the surface waves from this earthquake occurred worldwide^30^. Numerous previous studies have reported tremors/earthquakes triggered from the 2004 Sumatra–Andaman earthquake^31–34^.

Both minor and major arc rotations of Rayleigh waves can occur along the Earth’s perimeter, and the first (minor arc rotation) and second (major arc rotation) Rayleigh waves measured at the observation point are indicated as R1 and R2, respectively. Yoshizawa^30^ reported that the 2004 Sumatra earthquake in Indonesia generated numerous surface wave trains that travelled several times around the Earth. He extracted the long-period Airy phase of the fundamental-mode Rayleigh wave by applying a band-pass filter between 3 and 5 mHz. The long-period records displayed a clear series of multi-orbit Rayleigh waves that had circumnavigated the Earth more than six times, and they revealed clear signals of phases up to points R13–R14. In the 2011 Tohoku earthquake, Yomogida et al.^35^ showed clear phase signals at least up to R4, including multi-orbit Rayleigh waves, with a band-pass filter from 3 to 10 mHz.

A progressive surface wave propagating around the Earth has dispersibility properties. Dispersibility is observed when the propagation speeds of waves vary in accordance with their wavelength components, during which the initial waveforms gradually change with time. Surface waves indicative of dispersibility typically exhibit phase speeds that increase monotonically with several extrema across their wavelengths. Waves that correspond to a boundary between normal and inverse dispersions (that is, the extrema of the group velocity called the Airy phases) can propagate with large amplitudes. Rayleigh waves have vertical and radial components, and the wave amplitude decreases relatively slowly with distance, while the body wave amplitude decreases relatively rapidly. Oliver^36^ highlighted the dispersion relationships of Rayleigh and Love waves in continental and oceanic areas.

**Figure 1:**
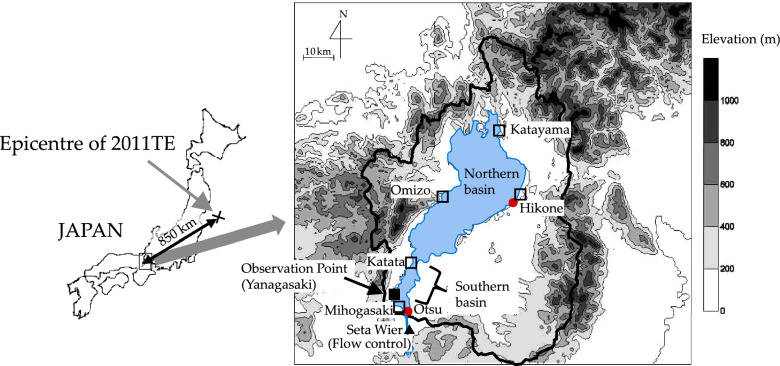
Mean water level (■) observation stations around Lake Biwa (dt = 60 s, 120 s). Water level observation sites were monitored by the Lake Biwa Work Office, Ministry of Land Infrastructure (□) (dt = 1 h), AMeDAS (Automated Meteorological Data Acquisition System) and Japan Meteorological Agency (●) (dt = 1 h), and water discharge data were provided by the Lake Biwa Work Office (dt = 1 h), Ministry of Land Infrastructure Waterworks Bureau, City of Kyoto, and the Kansai Electric Power Corporation (▲). The map was created using golden software Surfer 7 (Version 7.00, https://support.goldensoftware.com/hc/en-us/articles/228069868-Surfer-Version-History accessed on 23th September 2022) and a Digital Elevation Model (Geospatial Information Authority of Japan, https://www.gsi.go.jp/top.html accessed on 23th September 2022).

On 11 March 2011, an earthquake of magnitude 9.0 occurred off the Sanriku coast in the Tohoku area of Japan at 14:46 local time (UTC + 9). The epicentre was located in the Tohoku region (70 km east of Sendai City) in the Pacific Ocean at 38°6ʹ12″N, 142°51ʹ36″E (JMA). In the Lake Biwa region, approximately 850 km southwest of the epicentre (Fig. 1), average land displacements following the earthquake were 6 cm downward and 16 cm horizontal in the southeast direction (Geospatial Information Authority of Japan, GSI)^37^. Regarding the 2011 Tohoku earthquake (2011TE), many reports have suggsted remote triggering from the 2011 Tohoku earthquake^38–41^. Bondevik (2013) showed that S and Love waves were the main reason for the seiche in Norway; this indicates that seismic events can be induced by forced vibration^42^. Although numerous studies on seismic seiches have been conducted since the 1800s, definite research has not been performed on seismic seiche-related oscillations resulting from Rayleigh waves generated by large earthquakes. Understanding the effects of Rayleigh waves generated by large earthquakes will play a critical role in elucidating the propagation of seismic waves. The objective of this study was to identify the effect of Rayleigh waves on water level fluctuations in Lake Biwa after the 2011TE.

## Results

### Surface seiches of Lake Biwa

**Figure 2:**
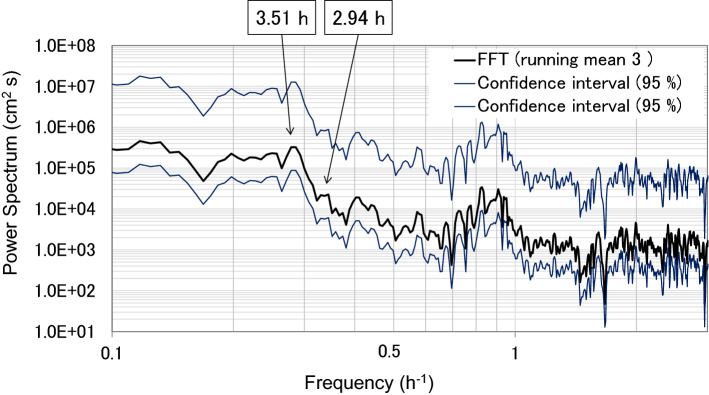
Fast Fourier transform (FFT) power spectrum of water level fluctuations measured at the Yanagasaki pier. The 4 h period enclosed with a circle corresponds to the surface seiche first mode in Lake Biwa.

In Lake Biwa, five modes of seiches have been identified by observation and simulation (Table A1). The amplitudes of the Lake Biwa surface seiches varied by approximately 0.06 m, which is the expected amplitude variation of lake seiches depending on wind speed and direction (Fig. A2). The period of the first mode of the surface seiche in Lake Biwa was calculated using the fast Fourier transform (FFT) method as approximately 4 h (Fig. 2), which is similar to the results of Imasato^16^ (Table A1).

### Time series of water levels of Lake Biwa after the Tohoku earthquake

**Figure 3:**
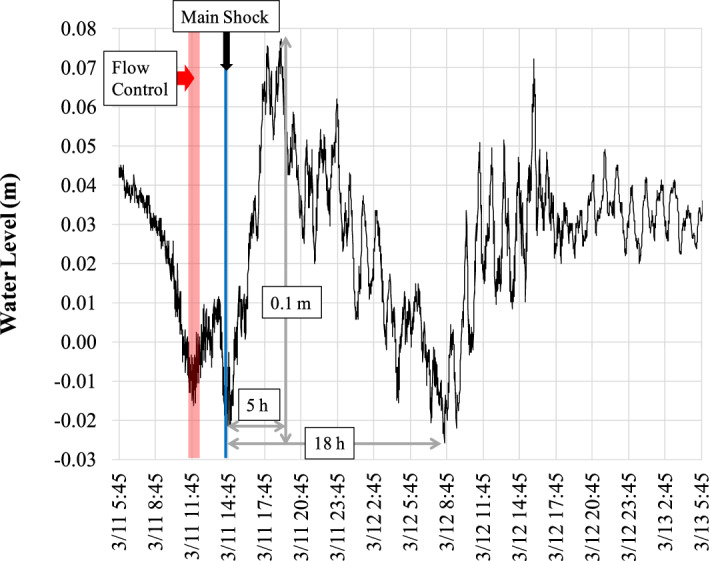
Time series of the water level measured at the Yanagasaki pier in the southern basin from 11 to 13 March 2011. Water discharge at the Araizeki weir was controlled from 11:40 to 12:40. The flow control time at the Seta Araizeki weir is indicated by the shaded bar. 2011TE occurred at 14:46 (main shock) on 11 March, causing the water level to increase for 5 h before returning to its original level 18 h later.

**Figure 4:**
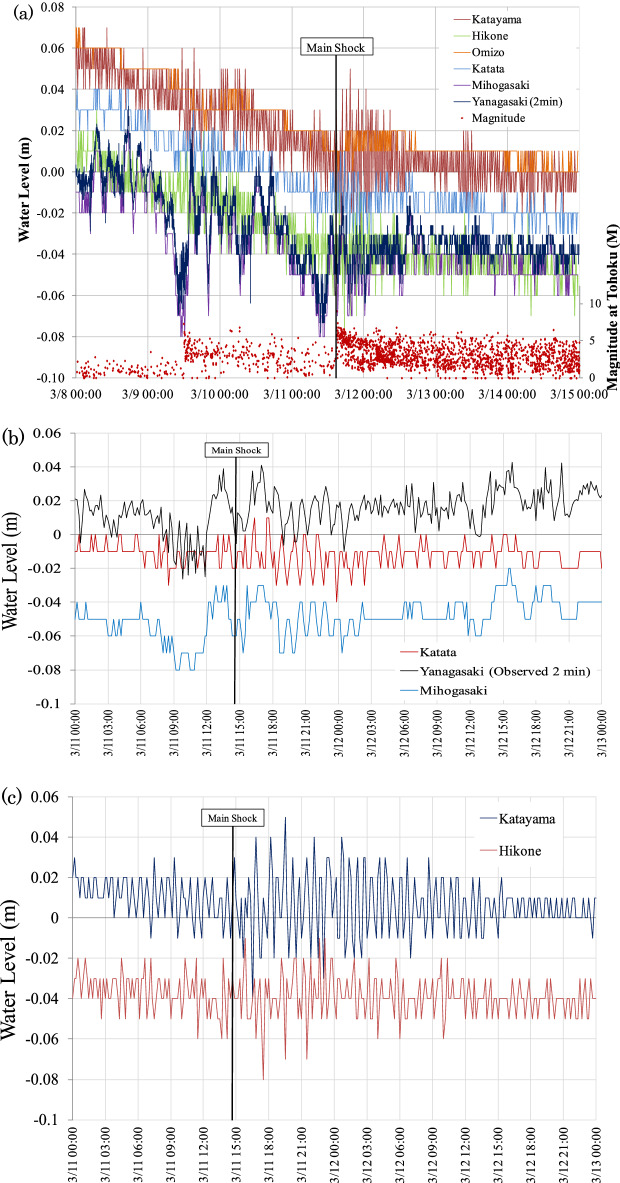
(**a**) Time series of water level changes measured at an interval of 10 min at five stations and an interval of 2 min at the Yanagasaki pier (Fig. 1) in Lake Biwa from 8 to 15 March 2011. The magnitude and frequencies of earthquakes in the Tohoku district are indicated by dots. (**b**) Time series of water level changes at Yanagasaki (2 min intervals), and Katata and Mihogasaki (10 min intervals) in the southern basin from 11 to 13 March 2011. (**c**) Time series of water level changes at Katayama and Hikone (10 min intervals) in the northern basin.

The outflow at the Seta Araizeki weir, where nine floodgates are used to control the discharge, decreased from 250 m^3^ s^−1^ before the earthquake to 120 m^3^ s^−1^ after the earthquake. Approximately 3 h before the earthquake occurred, the water level started to decrease and dropped to 0.03 m (Fig. 3). When the earthquake occurred at 14:46 local time (UTC + 9), the water level changed dramatically. After the earthquake, the water level increased by approximately 0.10 m (from − 0.02 m to + 0.08 m) within 5 h after the main shock. Following the peak at 22:00 on 11 March, water returned to its original level in approximately 18 h (Fig. 3). Similar water level changes were recorded at the north basin (Mihogasaki) monitoring location (Fig. 4a). The changes that occurred were highly complex and differed significantly between the shallow southern and deep northern basins of Lake Biwa (Fig. 4b,c). Overall, considerable variations in the water level were observed several times along with wavy motions until approximately 13:00 on 13 March.

Wind-caused seiches in Lake Biwa are typically generated by surface waters that are affected by strong winds at Hikone and Otsu (Fig. 1, Fig. A1). However, a weak wind occurred before the morning of 11 March; therefore, there was little possibility of the occurrence of surface seiche-related oscillation. Conversely, after 13:00 on 11 March (at the approximate time of the earthquake), a strong and dominant southwest wind was present (Fig. A4). These conditions were ideal for the development of a seiche with a dominant first mode. Overall, the earthquake generated a highly complex superposition of waves (Fig. 3).

### Spectral data

**Figure 5:**
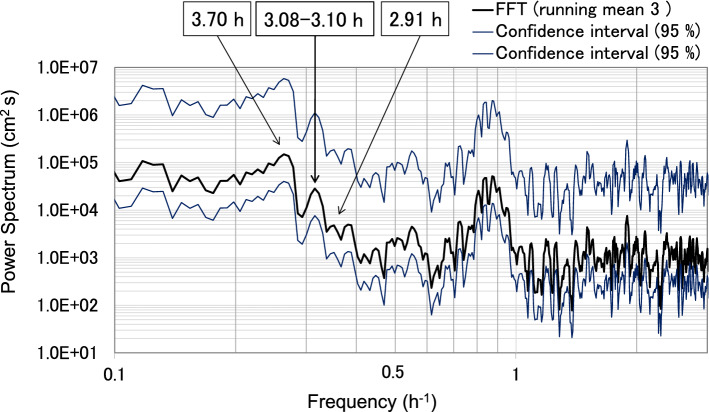
Spectra of water level fluctuations using FFT data at 2 min intervals measured at Yanagasaki, located at the southern end of the lake. N is the number of data points used for the spectral analysis. The calculation interval before the earthquake was from 20:44 5 March 2011 to 12:58 11 March 2011 (N = 4096), and the interval after the earthquake was from 13:00 11 March 2011 to 05:30 17 March 2011 (N = 4096).

Water level spectral analyses were conducted using both FFT and the maximum entropy method (MEM). The FFT analyses were categorised into two durations: before and after the 2011TE; for both cases, the number of datapoints (N) was 4096. The interval before the earthquake was from 10:14 on 5 March 2011 to 14:44 on 11 March 2011, and the interval after the earthquake was from 14:46 on 11 March 2011 to 07:16 on 17 March 2011(Figs. 2, 5). Each interval was separated into three distinct sections with 95% confidence intervals. We confirmed the presence of five typical surface seiche modes of Lake Biwa before and after the earthquake (Figs. 2, 5). The most prominent difference before and after the 2011TE was the existence of relatively high waves with a period of 3.08–3.10 h, which appeared only after the earthquake (Figs. 2, 5).

MEM spectral analyses were also performed with N = 1800. The interval before the earthquake ranged from 14:46 on 9 March 2011 to 14:44 on 11 March 2011, and the interval after the earthquake ranged from 14:46 on 11 March 2011 to 02:44 on 14 March 2011. The MEM spectral analysis also showed a spectral peak with a period of 3.08–3.10 h after the earthquake.

**Figure 6:**
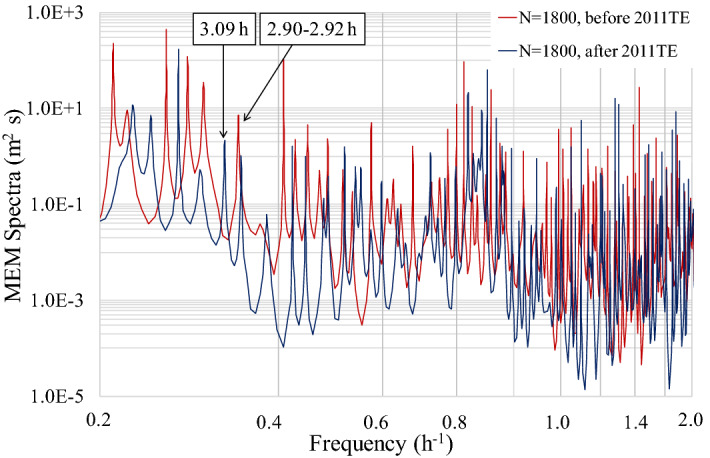
Maximum entropy method (MEM) spectra of water levels measured at the Yanagasaki pier in Lake Biwa before and after the 2011TE. N is the number of data points used for spectral analysis. The data before the 2011TE covers the period from 14:46 9 March to 14:44 11 March (N = 1,800), and the data after the 2011TE was obtained from 14:46 11 March to 02:44 14 March (N = 1800).

**Figure 7:**
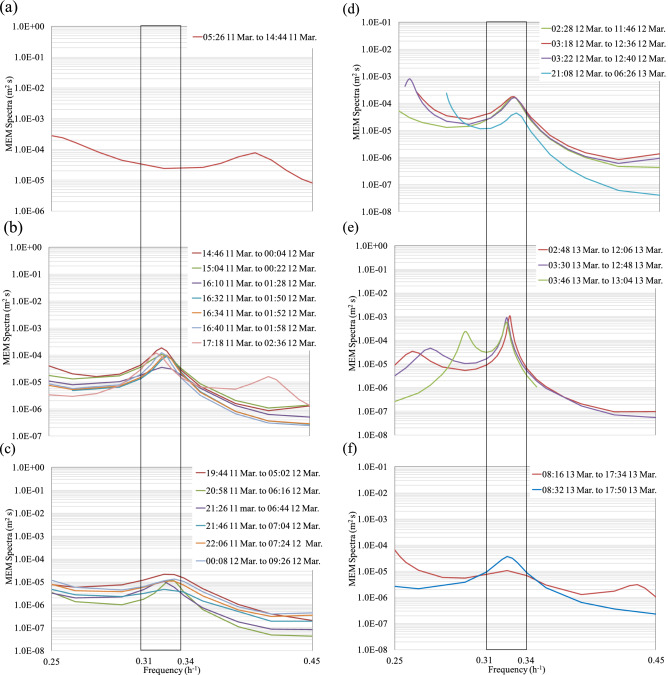
MEM spectra of water levels measured at the Yanagasaki pier in Lake Biwa before and after the 2011TE, where ‘N’ is the number of data points used for spectral analysis. The MEM spectra of the water levels focussed on the frequencies between 0.25 and 0.45 h^-1^. (**a**) MEM spectra of water levels using N = 280 increments before the 2011TE. No spectral peak was observed at a frequency of 0.32 h^-1^. The data before the 2011TE covered the period from 14:46 9 March to 14:44 11 March (N = 280). (**b**) MEM spectra of water levels using N = 280 increments after the 2011TE. Spectral peaks occurred at a frequency of approximately 0.32 h^-1^ as indicated by the shaded bar. The duration of this analysis was 14:46 11 March to 02:36 12 March 2011. (**c**) Analysis for the duration of 19:44 11 March to 09:26 12 March 2011. (**d**) Analysis for the duration of 02:28 12 March to 06:26 13 March 2011. (**e**) Analysis for the duration of 02:48 13 March to 13:04 13 March 2011. (**f**) Analysis for the duration of 15:20 13 March to 01:34 14 March 2011.

**Figure 8:**
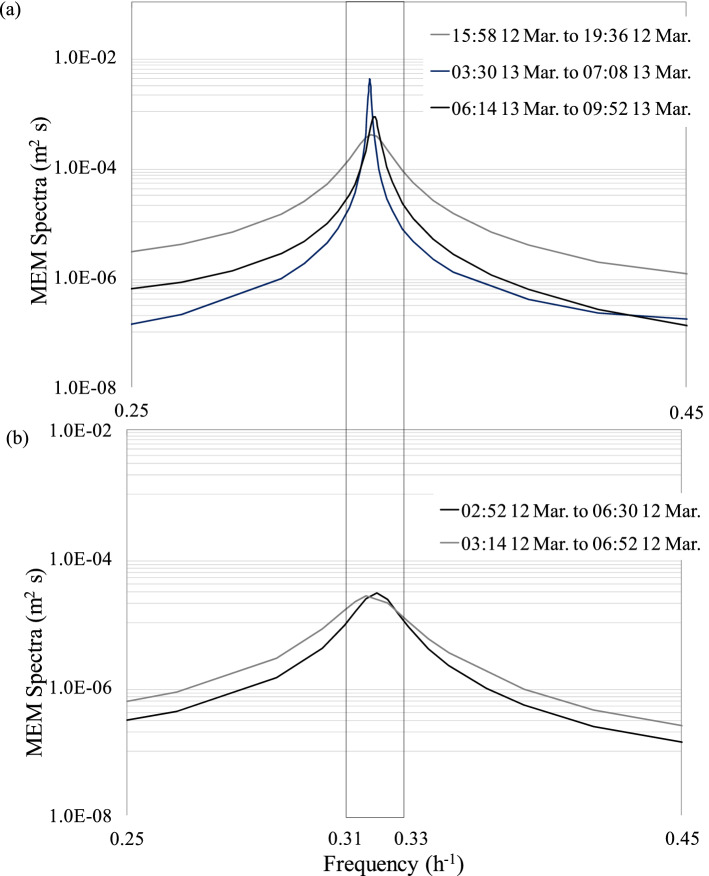
MEM spectra of water levels at a frequency of 0.32 h^-1^ using N = 110 increments after the 2011TE. (**a**) Data after the post-2011TE period were obtained for 02:52 12 March to 06:30 12 March and 03:14 12 March to 06:52 12 March 2011. (**b**) Data for the post-2011TE period were obtained for 15:58 12 March to 19:36 12 March, 03:30 13 March to 07:08 13 March, and 06:14 13 March to 09:52 13 March 2011.

Although the spectral peak with a period of 3.08–3.10 h was relatively weak, this peak was apparent in all the analyses using monitoring data points (N = 1800) after the 2011TE (Fig. 6). We conducted a search targeting the 3.08–3.10 h period, shifting the value of N = 1800 in increments of N = 280. Before the 2011TE, no peaks between 3.08–3.10 h were observed (Fig. 7a). After the 2011TE, the peaks showed some fluctuation, ranging between 3.08 h and 3.10 h (Fig. 7b,c). However, an obvious peak was observed approximately 24 h after the 2011TE (Fig. 7d–f). The N = 250 increments in Fig. 7a–f were more accurate than the N = 1800 increments, allowing us to determine the exact moment of appearance of the spectral peak with a period of 3.08 to 3.10 h (Fig. 7a–f). To further analyse the shaking and exciting characteristics of Rayleigh waves in the lake, we used 1800 monitoring data points with increments of N = 110 to calculate the MEM spectrum (Fig. 8a,b). This allowed for greater precision than the N = 280 increment and enabled us to determine the exact moment when the 3.08–3.10 h spectrum peaks appeared (Fig. 8a,b).

## Discussion

### Seismic seiche-related oscillation excited by Rayleigh waves

**Figure 9:**
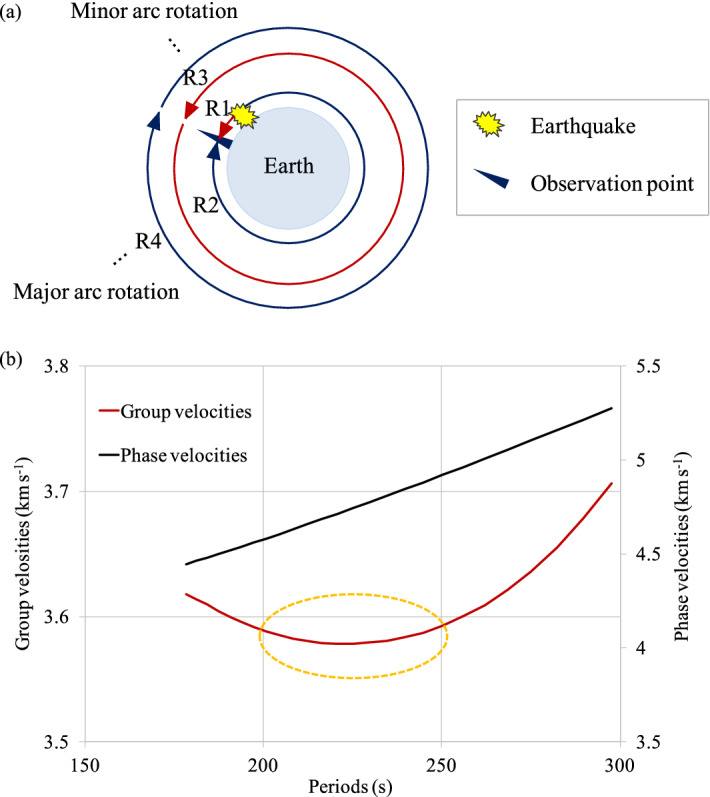
Illustration of Rayleigh waves and Rayleigh wave dispersion. (**a**) Illustration of the propagation of Rayleigh waves along the Earth’s surface. When an earthquake occurs, and Rayleigh waves are generated, the waves propagate along both the minor arc and major arc pathways around Earth. (**b**) Rayleigh wave dispersion modified from Oliver^36^, Kanamori^43^, and Dziewonski^44^. According to this model of dispersibility, the group velocity of a Rayleigh wave can be maximal near wave packets with a period of 240 s, which is approximately 3.6 km s^-1^^36^.

A surface seiche with a period of 3.08–3.10 h has never been observed in Lake Biwa (Table A1), and we deduced that this type of wave could have been generated by the 2011TE because no wave peaks with a period of 3.08–3.10 h appeared in the 1800 monitoring data points prior to the earthquake (from 02:46 on 9 March to 14:44 on 11 March 2011) (Fig. 6). After the 2011TE, scientists reported a global circulation of Rayleigh waves^30^. When a large earthquake occurs, Love and Rayleigh waves are generated as surface waves that propagate along both the minor and the major arcs from the epicentre of the earthquake around the Earth (Fig. 9a). According to the observed Rayleigh wave dispersibility, the group velocity of the Rayleigh waves (approximately 3.60 km s^−1^) was likely the highest near wave packets with a period of 240 s (Fig. 9b). By setting the group velocity of the 240 s period Rayleigh waves to approximately 3.6 km s^−1^, we could establish that the travel time of the Rayleigh wave at approximately 240 s for an angular distance of 360°, i.e. the great circle of the entire globe, which is a great circle around the entire earth, is 11,113 to 11,131 s (approximately 3.09 h). This period is remarkably similar to the period of 3.08 to 3.10 h observed in Lake Biwa which corresponds to the seiche period observed in Lake Biwa after the 2011TE.

In summary, from 12 to 14 March 2011, under weak wind conditions, the water level of Lake Biwa showed oscillations with a period of 3.08–3.10 h following 2011TE. This oscillation period of 3.08–3.10 h is very different from the natural oscillation of Lake Biwa, which indicates that the oscillation was resonantly generated in Lake Biwa by the 2011 TE. We could establish that the oscillation period corresponded to the seiche period observed in Lake Biwa after the 2011 TE. Therefore, the observed water level changes in Lake Biwa after 2011TE indicated that the waves with a period of 3.08–3.10 h were forced by Rayleigh waves, characterized by a frequency close to the natural frequency, which were excited by the earthquake.

We now discuss why the Love wave did not excite the seiche. The group velocity of Love waves is faster than that of the Rayleigh wave and travels the entire globe for approximtely 2.5 h; however, the surface waves along the major and minor arcs of the great circle from the Tohoku earthquake propagate in the NE–SW direction. The Rayleigh and Love waves polarise in the longitudinal and transverse directions, i.e. NE–SW and NW–SE directions, which correspond to the major and minor axes of the elliptical shape of Lake Biwa (Fig. 1), respectively. Assuming the broader width of the lake is more resonant with the seismic ground motion to trigger seismic seiche, Rayleigh waves can more effectively function in triggering than Love waves.

The same principle applies in this case. On 9 May 2010, a 7.3 magnitude earthquake occurred near Sumatra at 14:59 local time (UTC + 7). The water levels in Lake Biwa, which is located approximately 5000 km away from the epicentre of this earthquake, were measured at 60 s intervals using the same capacity-type water level gauge. The water level changed considerably, exhibiting an amplitude of 15–30 mm after the main shock of the earthquake (Fig. A4a). The periods in the same duration were calculated by FFT (used C, N = 4096, dt = 60 s) shown in Fig. 4a,b.

## Conclusions

This study analysed the complex water level changes in Lake Biwa created by the 2011TE using FFT and MEM spectral analyses. The results of both methods revealed previously unobserved waves with a duration of 3.08–3.10 h after the 2011TE; these waves were relatively weak under weak wind conditions. The duration of these waves satisfied the dispersion relationship of the Rayleigh waves with a 3.08–3.10 h period that were excited by the 2011TE. A subsequent MEM spectral analysis for 110 data segments showed no 3.08–3.10 h period peaks before the 2011TE and definite peaks after the 2011TE. Therefore, we deduced that the 3.08–3.10 h period waves were excited by the forced oscillation of Rayleigh waves characterised by a frequency similar to the natural frequency and a 3.08–3.10 h period after the 2011TE.

The amplitude of the seismic seiche-related oscillation excited by the forced oscillation from the Rayleigh waves was approximately 20 mm. Although no changes occurred in the water volume physically, this amplitude could have a major impact on the ecosystem in the littoral region of the lake. Additionally, water level fluctuations in a lake can be amplified by forced oscillation from Rayleigh waves and are a good indicator of earthquakes with magnitudes > 7.0, which may produce Rayleigh waves and an elevated tsunami.

## Methods

### Observation site

The surface area and mean water depth of the northern basin are 618.65 km^2^ and 43 m, respectively, and the respective values of the southern basin are 51.6 km^2^ and 4 m^45^. The study site was the Yanagasaki pier located at the southwest end of the South Basin. The elevation of the site is 87 m above sea level, and the mean depth is 2 m (Fig. 1). The pier is typically not busy, and boat arrivals and departures typically occur for several minutes twice a day; thus, their effect on the water column is marginal.

### Measurement of water level fluctuations

To determine water level fluctuations in Lake Biwa, we deployed a capacity-type water level gauge at the end of the Yanagasaki pier (Fig. A3). This instrument measured water level changes using a linear relationship between the water level and a resistance–capacitance oscillator, which was developed by connecting two wires (Teflon and stainless-steel wires) in parallel. The resolution of the water level gauge was 1 mm, and the accuracy was ± 2 mm. A HIOKI data logger was used to record water level fluctuations with sampling intervals of 1 min (from 22 April to 20 May 2010 and from 1 November 2011 to 31 March 2012) and 2 min (from 21 May 2010 to 31 October 2011).

### Other data acquisition

We measured the water level at a single point in the southwest region of Lake Biwa and used additional water level data measured at five stations operated by the Biwako Office of the Ministry of Land Infrastructure, namely: Katayama, Omizo, Hikone, Katata and Mihogasaki (Fig. 1). Data on artificially controlled discharge through the Seta River were also provided by the Biwako Office. Nine floodgates controlled the flow at the Seta Araizeki weir. Additional discharge data for the two canals and the hydropower station channel were provided by the Waterworks Bureau in Kyoto city, and the Kansai Electric Power Corporation, respectively. Moreover, meteorological data and earthquake information were provided by JMA, Geospatial Information Authority of Japan (GSI), and United States Geological Survey (USGS).

### Spectral analysis

We used two different spectral analysis methods to identify characteristics of various types of surface waves. The FFT algorithm is a conventional method for analysing oscillatory motions to extract wave spectra, including surface seiches, under the assumption of repeating infinite length data instead of obtaining data for the given time series^46,47^. MEM was established as a spectral analysis technique by Burg^48^ to obtain wave spectra using short-period data for phenomena, such as seismic waves, because it can help perform spectral estimations to maximise information entropy with an autocorrelation function^49,50^.

## Supplementary Information

Supplementary Figure 1.

Supplementary Figure 2.

Supplementary Figure 3.

Supplementary Figure 4.

Supplementary Table 1.

## Data Availability

The datasets used and/or analysed during the current study available from the corresponding author on reasonable request.
