# Supplementary material for: Seismic seiche-related oscillations in Lake Biwa, Japan, after the 2011 Tohoku earthquake
Source: Sci Rep. 2022 Nov 11;12:19357. doi: 10.1038/s41598-022-23939-7 (PMC9652454; doi:10.1038/s41598-022-23939-7)
Supplement: Supplementary file 1 — Supplementary Figure 1. [file 41598_2022_23939_MOESM1_ESM.pdf]

Figure A1

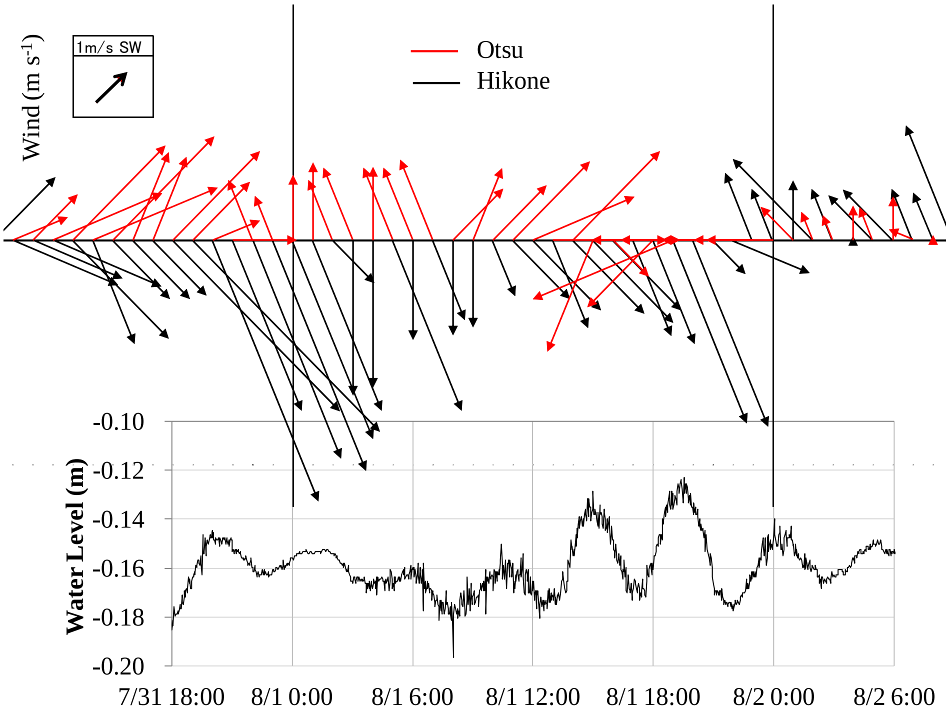

Fig. A1. Time series of the wind vectors measured at the Otsu Meteorological station (top panel) and water level changes at the Yanagasaki pier in the southern basin (bottom panel). The vertical lines indicate midnight on 1 and 2 August 2011.
