# Supplementary material for: Seismic seiche-related oscillations in Lake Biwa, Japan, after the 2011 Tohoku earthquake
Source: Sci Rep. 2022 Nov 11;12:19357. doi: 10.1038/s41598-022-23939-7 (PMC9652454; doi:10.1038/s41598-022-23939-7)
Supplement: Supplementary file 2 — Supplementary Figure 2. [file 41598_2022_23939_MOESM2_ESM.pdf]

Figure A2

Otsu

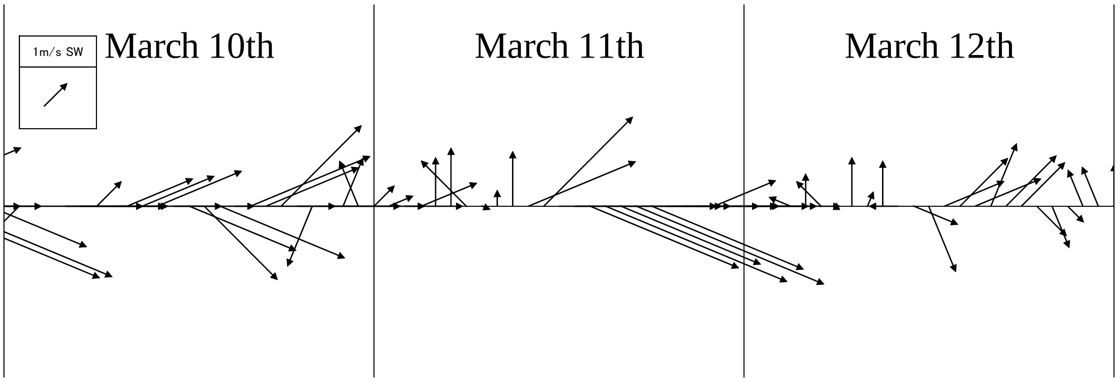

Hikone

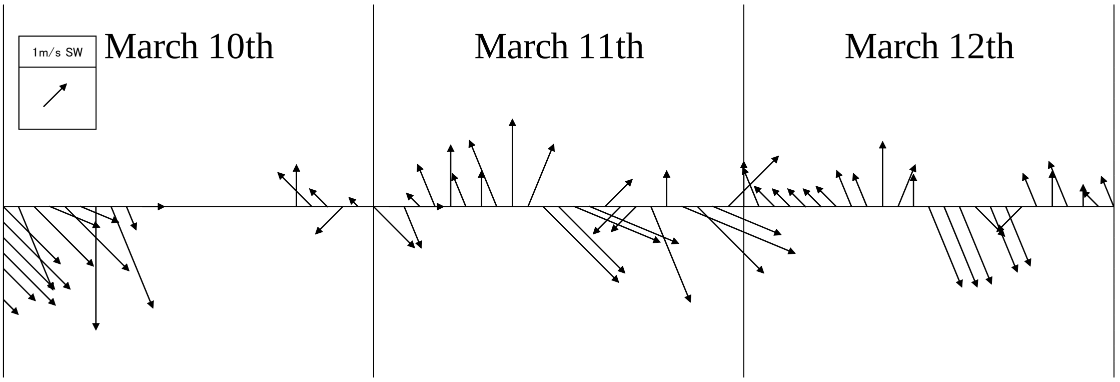

Fig. A2. Hourly vector diagram of wind speed and direction measured by AMeDAS at Otsu and Hikone in the Shiga prefecture from 00:00 8 March to 00:00 14 March 2011.
