# Supplementary material for: Seismic seiche-related oscillations in Lake Biwa, Japan, after the 2011 Tohoku earthquake
Source: Sci Rep. 2022 Nov 11;12:19357. doi: 10.1038/s41598-022-23939-7 (PMC9652454; doi:10.1038/s41598-022-23939-7)
Supplement: Supplementary file 3 — Supplementary Figure 3. [file 41598_2022_23939_MOESM3_ESM.pdf]

Figure A3

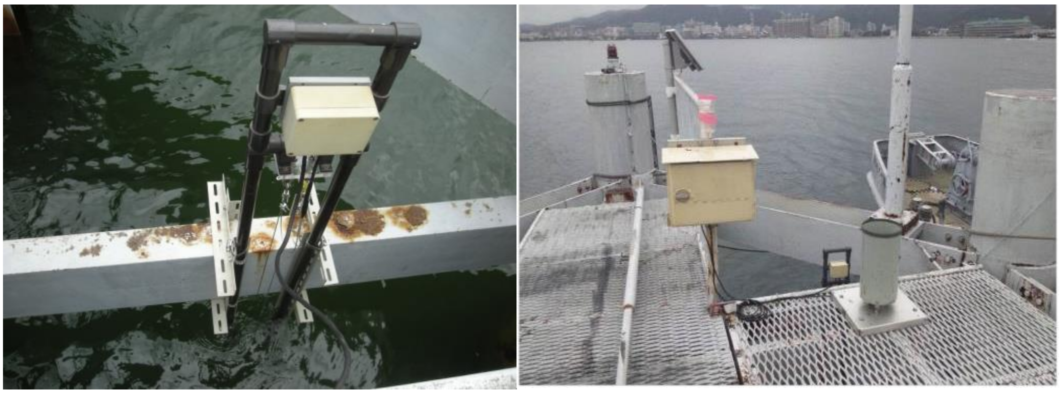

Fig. A3. Photos of the water level gauge (left) and observation site (right) with rain and water level gauges deployed at the Yanagasaki pier.
