# Supplementary material for: Seismic seiche-related oscillations in Lake Biwa, Japan, after the 2011 Tohoku earthquake
Source: Sci Rep. 2022 Nov 11;12:19357. doi: 10.1038/s41598-022-23939-7 (PMC9652454; doi:10.1038/s41598-022-23939-7)
Supplement: Supplementary file 4 — Supplementary Figure 4. [file 41598_2022_23939_MOESM4_ESM.pdf]

Figure A4

(a)

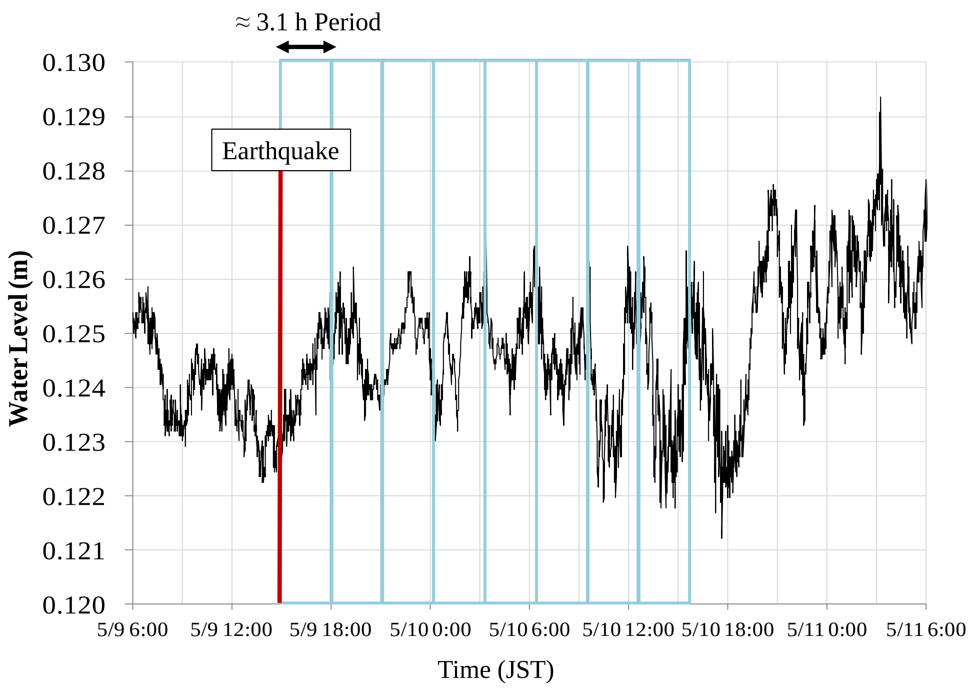

(b)

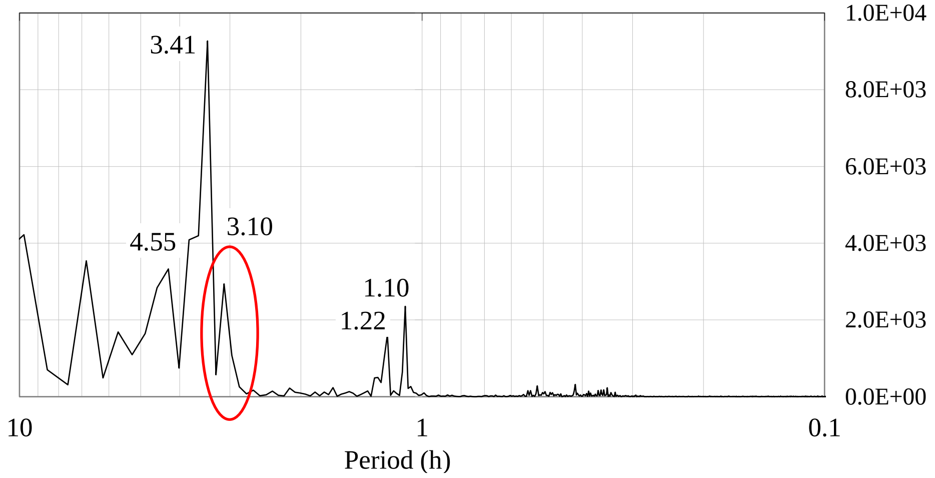

Fig. A4. The water level changes measured at an interval of 1 min at the Yanagasaki pier (Fig. 1) in Lake Biwa from 8 to 15 March 2010. On 9 May 2010, a 7.3 magnitude earthquake occurred near Sumatra at 14:59 local time (UTC+7). The coordinate time of

x axis is JST (UTC+9). (a) Time series of water level changes. (b) Spectra of water level fluctuations using FFT.
