# Supplementary material for: Seismic seiche-related oscillations in Lake Biwa, Japan, after the 2011 Tohoku earthquake
Source: Sci Rep. 2022 Nov 11;12:19357. doi: 10.1038/s41598-022-23939-7 (PMC9652454; doi:10.1038/s41598-022-23939-7)
Supplement: Supplementary file 5 — Supplementary Table 1. [file 41598_2022_23939_MOESM5_ESM.pdf]

Table A1 Previous studies on the surface seiches of Lake Biwa[16].

Investigator	Year		Period (min) Mode-Ⅰ	Mode-Ⅱ	Mode-Ⅲ	Mode-Ⅳ	Mode-Ⅴ	Others
Nakamura & Honda	1911	Obs.	231.2	72.6			30.5	25.2, 22.7
		Ex.						
Suda et al (Kobe Mar. Observ.)	1926	Obs.	242.0	71.0		36-37	30.0	25–22, 15
Takaya	1931	Obs.	236.2					
Nomitsu	1935	Obs.	250.0	68.4			30.0	20, 12, 5
Takahashi	1935	Cal.	208.0	68.0				
Nomitsu et al.	1937	Obs.						18–15, 5
Toyohara & Habu	1938	Obs.		66.0			32.0	
Takahashi & Namekawa	1938	Obs.	220.0					
Imasato	1970	Cal.	212.0	71.3	61.0	37.0	32.3	
Imasato	1971	Cal.	255.5	79.8	69.1			
		Obs.	243.9	74.1	65.1			
Imasato	1972	Cal.				38.7	31.9	51.1
		Obs.	232.3	74.1	66.4	40.2	32.6	
Imasato	1973	Obs.	229.8	72.7	65.1	40.1	30.5	
Mean		Obs.	229.8±12.2	72.2±2.2	65.1±1.5	40.1±0.6	30.5±0.8	
		Cal.	255.5	79.8	69.1	38.7	31.9	

Obs. = Observation, Ex.=Experiment, Cal.= Calculation
